# “‘Keep the Fire Burning’: Applications and Culturally Congruent Research Strategies for Research on Sexual and Reproductive Health With Indigenous Women”

**DOI:** 10.35844/001c.94023

**Published:** 2024-04-01

**Authors:** Jessica Liddell, Catherine McKinley, Amy Stiffarm

**Affiliations:** 1 School of Social Work, University of Montana; 2 School of Social Work, Tulane University; 3 Department of Indigenous Health, University of North Dakota School of Medicine & Health Sciences

**Keywords:** Indigenous, qualitative research, Native American, reproductive health, qualitative description, life-history interviews, cultural humility

## Abstract

Because of the long history of exploitative research with Indigenous groups, an ethical and empirical imperative exists for researchers, especially non-Indigenous scholars, to reflect on their own positionality and to use culturally congruent methodologies and strategies when conducting research. A simultaneous need is for research on the reproductive and sexual health experiences of Indigenous women, who experience extensive reproductive health disparities and reproductive injustices. The purpose of this article is to provide an example of a best practice in conducting community engaged research to explore the reproductive and sexual healthcare experiences of Indigenous women, through the identification of factors that promote and that act as barriers to health. We first provide an overview of the literature describing the history of researcher exploitation before providing an in-depth discussion of the methodology used in this study. We then describe an application of the qualitative description methodology in 31 semi-structured life-history interviews with Indigenous women. The strategies used in this study facilitated the provision of rich qualitative information about reproductive health, which will be used to develop interventions for tribal members. This study addresses gaps by providing an example of a culturally appropriate methodology and its application with Indigenous women.

## Introduction

The purpose of this article is to discern best practices for conducting community-engaged research through applying the *Toolkit for Ethical and Culturally Sensitive Research* with Indigenous peoples; we emulate how to center and integrate Indigenous Knowledge while conducting rigorous and ethical sexual violence and reproductive health research with Indigenous communities (Burnette et al., 2014). To this end, the article is situated within the context of a specific study, investigating reproductive and sexual health experiences of Indigenous women in a Gulf Coast Indigenous tribe. The lead author chose this focus area to develop interventions that center Indigenous women’s health holistically. Though impact-based research is important, this current article aims to explicate a methodological approach for replication and application to other topics in Indigenous research. Given the extensive context of exploitative research that is part of a broader history of historical oppression, models and demonstrations of positive research strategies are needed (Ball, 2005; Begay et al., 2019; Burnette et al., 2014; Burnette & Sanders, 2014; Cochran et al., 2008). This methods article describes specific research approaches, theories, and principles and provides valuable background surrounding the need for ethical and community-engaged research with Indigenous tribes. Contextualizing these methods and demonstrating applicability is especially important to the authors’ goal of contributing valuable and usable information to be considered in the broader discourse of equitable research.

In this work, we explain the importance of the researcher’s positionality and demonstrate how key elements of cultural competence, humility, and cultural safety, guiding theories and principles for the study, were applied during the research process, including research design, data collection and analysis, and dissemination. We apply the steps outlined by Burnette et al. (2014) in their *Toolkit for Ethical and Culturally Sensitive Research* for conducting rigorous and ethical research with Indigenous communities were especially helpful for ensuring adherence to these guiding theories and principles (Burnette et al., 2014). We discuss the overall design of community-based research (CBR), its applicability to research in Indigenous contexts, specifics of the qualitative descriptive approach, and the rationale for using this method. We also provide information on the steps taken to honor participants’ autonomy and the tribe’s desires for the study. Data disclosed in this article are not comprehensive but are specific for demonstrating the iterative process of reflection and action in research, carried out in coordination between the first author and Indigenous participants. The authors believe this methods article presents applicable, specific, and empirically based considerations for research in Indigenous contexts. This article extends and applies the work of Burnette et al. (2014), whose toolkit for ethical and culturally sensitive research to sexual and reproductive health research with Indigenous peoples. In its application, we provide a roadmap for Indigenous and non-Indigenous scholars working in solidarity in the spirit of the relevance, reciprocity, respect, and shared responsibility (Kirkness & Barnhardt, 1991).

### Indigenous Context

We recognize important differences exist in culture and traditions among distinct tribes and acknowledge that this research is situated within the context of work alongside one particular tribe. Relevant background into the tribe’s context is provided; however, the identity of this tribe will remain confidential in published material in accordance with tribal agreements and guidelines for culturally sensitive research with Indigenous peoples (Burnette et al., 2014). We strive to acknowledge the multiplicity of Indigenous communities while providing an example of thoughtful research methodologies in application, not simply as theory.

The study referenced in this article was carried out with the participation and collaboration of a state-recognized tribe in the U.S. Gulf South region. The tribe consists of an estimated 17,000 members which continues to experience large environmental shifts. The Gulf Coast is particularly vulnerable to land loss and increased hurricanes, flooding, and coastal storms associated with climate change. Additionally, the areas’ wetlands and waterways that have been home to this tribe and provide important sustenance have been deeply impacted by industry. A complex relationship exists between industry and environmental concerns, as many tribal members are employed by the region’s oil production and water management companies.

The tribe discussed in this article, and other tribes in the Southeastern region of the United States, have been disproportionately impacted by barriers to receiving federal recognition (National Conference on State Legislatures, 2020; Salazar, 2016), which affected their political autonomy and stymied access to federal resources and benefits. Without federal recognition, the tribe has been prevented from accessing services provided by federal agencies like Indian Affairs and the IHS, hindering the provision of healthcare and social benefits (Crepelle, 2018; Fitzgerald, 2015). Furthermore, though this tribe is state-recognized, state tribal recognition does not confer the same relationship between the federal government and Tribal Nations, impacting governmental recognition of their sovereignty (Crepelle, 2018; Fitzgerald, 2015). The process of applying for and receiving federal recognition has been described as complex and ever-changing, with multiple standards used to determine a tribe’s eligibility (Crepelle, 2018; Fitzgerald, 2015; Fletcher, 2006). Tribes that were not designated reservation land during the Indian Removal Act have faced additional barriers to receiving federal recognition, as there is a frequent lack of government documentation “proving” tribal affiliation and membership (Crepelle, 2018; Fitzgerald, 2015; Fletcher, 2006).

Despite experiencing obstacles to federal recognition and environmental threats, this tribe maintains many cultural traditions and values, such as family closeness, advocating for others, generosity, and self-sufficiency. In using a strengths-based research approach with this tribe, we sought to highlight tribal resiliency and decenter settler-colonialism. Focusing on strengths is paramount for acknowledging agency, avoiding portrayals of passive victimhood, and producing knowledge that is solution-oriented and does not simply document disparities (Burnette et al., 2014; Creese & Frisby, 2011; Davis, 2003; Green & Haines, 2016; Gurr, 2014; Minkler & Wallerstein, 2011; Strand et al., 2003).

We would like to make an important note on terminology. Many Indigenous languages recognize several different genders and sometimes use inclusive terms, such as birthing people, to describe those capable of getting pregnant and giving birth. We feel strongly about the need to honor the complexity of gender in language and to account for multiple gender identities in research. However, the words “woman” and “women” are used throughout this article because study participants self-identified as women. Additionally, most referenced literature uses data specific to those who identify as women or have been labeled as women. To best represent this data, we use the gender identifiers used in the original research articles.

## Critique of Conventional Research Methods

Because research itself is a social activity informed by social conditions and power relations, all research activities are political (Strega & Brown, 2015). Research has been used as a tool to perpetuate societal inequality, oppression, stereotypes, and dominance over marginalized groups, including Indigenous communities (Denzin et al., 2008; Roberts, 1999; Smith, 1999). Conventional research, which has generally been presented as offering a “neutral observer” view of the world, is critiqued for ignoring how “science has been used as a tool to carve out the lines of normalcy on multiple bodies” (Manning, 2015, p. 205). Research has then been used to inform economic and social policies and justify government regulation of social services, including education, health, and social welfare programs (Carroll et al., 2019; Kovach, 2010).

Exploitative research is not just a part of research history in the long past. As recently as 1989, a team of researchers from Arizona State University took blood samples from the Havasupai tribe under the guise of investigating diabetes. However, researchers then tested the blood for schizophrenia and other mental health disorders and allowed the blood samples to be distributed nationally to other researchers (Cochran et al., 2008). For tribes struggling to gain federal recognition, this type of bait-and-switch research can have real consequences. For example, blood taken from the Canadian Nuu-chah-nulth people (i.e., under the guise of arthritis research) was then used to establish tribal/nontribal ancestry, among other projects without their consent (Cochran et al., 2008).

Community exploitation is not isolated to medical or biological research, which can be seen in the many ethnographies of Indigenous and other “exotic” cultures (Kovach, 2010; Smith, 1999). Anthropology and sociology research have also been used to justify the removal of Indigenous children from their parents and for taking control of natural resources (Mertens et al., 2013). These ethnographies provided some of the “evidence” needed to support the removal of Indigenous children from their families to place them in boarding schools (Kovach, 2010).

This example illustrates how governmental reproductive health policies have generally served to reproduce existing social hierarchies, discourage the reproduction of marginalized groups, and encourage the reproduction of privileged women (Bell, 2014; Davis, 2003; Flavin, 2008; Hill Collins, 2006; Kluchin, 2011; Roberts, 1999).

Researcher findings have frequently been used to justify the control and restriction of women’s reproductive autonomy (Gurr, 2014; Kovach, 2010; Smith, 1999). This history of exploitation makes many marginalized groups of women hesitant to participate in research projects (Parker, 2017; Smith, 1999). Special care is needed when researching Indigenous sexual and reproductive health specifically because social institutions have consistently monitored and restricted Indigenous women’s sexual and reproductive decisions (Ginsburg & Rapp, 1991). This highlights the important need for empowering and community-driven research in reproductive health (Gurr, 2014).

At one end of the continuum, research is exploitative and extractive; even seemingly innocuous research can inadvertently cause harm or fail to contribute meaningfully to those studied. Some contemporary research continues to be problematic for many Indigenous peoples because of the types of implications drawn from its findings (e.g., deficit framings, or focus on disparities), the use of culturally incongruent methods, and a general lack of responsiveness to the concerns of the community (Cochran et al., 2008; Hyett et al., 2019). In avoiding research with Indigenous participants altogether, Indigenous communities are excluded from valuable innovations, particularly in health research (Begay et al., 2019).

A growing body of research has been conducted via collaborations between non-Indigenous and Indigenous researchers with a focus on using equitable and effective methods of inquiry (Carlson, 2017; Parker et al., 2019). For example, a research project investigating the Gold King Mine Spill used the concept of two-eyed seeing to conduct focus groups culturally adapted to the Diné tribe (Teufel-Shone et al., 2021; Van Horne et al., 2023). Two-eyed seeing is the theoretical framework in which Indigenous and non-Indigenous worldviews are concurrently referenced and honored (Martin, 2012). An important aspect of this conceptual framework is an equitable valuing of worldviews respective to the tools and information they provide (Martin, 2012). Additionally, ethics trainings and ethical guiding bodies (e.g., tribal Institutional Review Boards [IRBs]) have been created by or in coordination with Indigenous communities to ensure more culturally congruent research practices are enacted and to safeguard against explosive research (Carroll et al., 2022).

Research approaches that center the community’s autonomy throughout the research process may be one way to conduct meaningful studies that avoid repeating oppressive practices (Parker, 2017). Given a broad context of historical oppression in research, more examples, models, and information on how to conduct community-engaged, culturally sensitive, and ethical research is needed. These examples and models can inform best practices and norms when working with Indigenous peoples. Because of its political and situated nature, choices about approach and methodology must be informed by thoughtful reflection about the social, political, and cultural context of the research project.

## Importance of Researcher Positionality

The researcher’s theoretical and philosophical orientation toward research is of central importance in conducting ethical and culturally congruent research. Intensive reflection throughout the research process is fundamental and extends to the research project, the researcher, and the researcher’s orientation to research itself (Strega & Brown, 2015). A researcher informed by their ontological and epistemological views positions themselves in relation to research (Strega & Brown, 2015). Strega and Brown (2015) defined ontology as a researcher’s worldview and defined epistemology as the researcher’s philosophy about what counts as knowledge. Social-justice-oriented approaches to research challenge the types of methods used and the philosophical foundations of research itself (Strega & Brown, 2015). Many Indigenous scholars are critical of researchers who do not fully analyze their intersectional positionality (Burnette et al., 2014; Kovach, 2010; Smith, 1999; TallBear, 2014).

A researcher should first undergo a deep reflection of the researcher’s positionality. This process is necessary throughout the entire research project and cannot simply occur at project onset. This concept was also emphasized by in critical thought, leader, Paolo Freire (2000) who believed working in solidarity requires constant self-examination and reflexivity. This practice should entail an “uncomfortable reflexivity” that allows reflection to be “messy, confessional, and tentative” (Strega & Brown, 2015, p. 12). Strega and Brown (2015) cautioned against reflexivity that only focuses on the researcher’s personal biography without attending to larger social and political forces.

Without acknowledging these larger power dynamics, there is risk that a researcher’s whiteness will become the focus of detriments to positive impact and reflection (Strega & Brown, 2015). Failing to acknowledge structural inequalities is especially problematic because, as Strega and Brown (2015) noted, “simply listing our biographical or personal information may even serve to establish and assert our authority” (p. 10). Throughout this project, the lead author has engaged in reflection with themselves and their coauthors about their role as a middle-class, White, non-Indigenous researcher from another region and who, at the time of this research, was a student at a prestigious private university located in a large city.

As a non-Indigenous woman, the lead author has examined her theoretical and methodological research orientation and values, intentionally framing research questions. Though investigating reproductive needs and gaps in access is essential for achieving just reproductive health outcomes, the lead author has been drawn to strengths-based approaches to research. In addition to documenting needs, we have included an analysis of the tribal community’s strengths and have highlighted how individuals have acted to promote their reproductive health.

The second author has been working with Indigenous communities for more than a decade and has focused on women’s health, violence prevention, health equity, and resilience within a context of historical oppression. The second author is deeply dedicated to contributing to improved health equity for Indigenous peoples and seeks to work in solidarity with these peoples in decolonizing, emancipatory projects. As a non-Indigenous White woman, the second author learned from Indigenous and non-Indigenous researchers how to work in culturally sensitive and ethical ways, given her positionality (Burnette et al., 2014). The second author has mentored the lead author to work in relational reciprocity within partnered communities. Thus, this toolkit for ethical sensitivity frames the approach and methodology of this research. After the second author had completed an extensive study with the focal community (McKinley et al., 2019), the lead author gained entry with the same tribe by following the recommendation to “get invited” and “build a positive reputation” through a “long-term” commitment with the tribe (Burnette et al., 2014, p. 11).

The third author is an Indigenous woman and Indigenous health researcher. The third author is an enrolled Tribal Member within the Fort Belknap Indian community. The third author is also descended from the Cree and Blackfeet Nations. The third author has a deep understanding of Indigenous epistemology, ontology, and worldview and was invited as a contributor due to specific training and experience working with Indigenous communities. The third author approaches research questions with an Indigenous epistemology, where Indigenous traditional knowledge has value, just like Western-based research. The third author’s ontology includes the belief of holism — that all beings are interconnected and rely on each other for existence. The third author’s axiology is based on their relational responsibility to their community and other Indigenous populations. As an Indigenous researcher, the third author commits their work to embody respect, relevance to Indigenous communities, reciprocity, and responsibility when working on projects relevant to Indigenous populations.

## Guiding Theories and Principles for CBR

Best practices for conducting community-engaged research can be gleaned through applying the *Toolkit for Ethical and Culturally Sensitive Research* with Indigenous peoples, which enables the integration of Indigenous Knowledge into rigorous and ethical sexual violence and reproductive health research (Burnette et al., 2014). Concepts of cultural humility, competence, and safety are central to the research approach discussed in this article. One key principle of cultural competence includes reflection and awareness of one’s limitations (Lynch & Hanson, 2004). Congruent with the importance of continuous self-reflection, cultural competence does not refer to a specific set of skills but instead includes a variety of techniques and focuses on self-awareness in diverse contexts (Lynch & Hanson, 2004). As with situating one’s positionality in relationship to research, self-awareness and reflection are key (Lynch & Hanson, 2004). Additional principles include researcher openness, respect for other cultures, and a desire to value community strengths as resources (Lynch & Hanson, 2004).

Cultural humility requires researchers to have a humble orientation toward the research process by acknowledging they will inevitably make mistakes and being willing to adapt and own up to them (Gaudry, 2015). Humility also calls on researchers to acknowledge the immense expertise and knowledge held by participants. Finally, in situating themselves, researchers should take primary responsibility for their self-education and not burden study participants or the community with this education (Gaudry, 2015). The entire research process requires intense self-reflection and communication with the community, research team, and colleagues.

Though less frequently referenced, cultural safety is also important for guiding research (Curtis et al., 2019; Israel et al., 2005). A focus on cultural safety means honoring the community’s autonomy to decide which research activities and interactions facilitate feeling safe (Israel et al., 2005). Cultural safety means acknowledging the common power differential between the researcher and the researched, and knowing that community members may frequently not feel safe about collaboration (Israel et al., 2005). This combination of cultural humility and cultural safety with the procedures, process, and methods of CBR contrasts strongly with conventional research models (Israel et al., 2005).

## Application of CBR

CBR served as the overarching approach to the exemplary study on reproductive and sexual health experiences of Indigenous women. Separate from the many ethical reasons to perform research conducted with community input and collaboration, an extensive body of research has suggested CBR methods provide data of better quality and are more representative of actual community needs and desires (Beckman & Long, 2016; Israel et al., 1998; Minkler & Wallerstein, 2011; Parker, 2017; TallBear, 2014). This research may be more rigorous and practical because it incorporates local knowledge and is “based off the lived experience of the people involved” (Israel et al., 1998, p. 81). Using CBR to better understand community contexts creates a powerful mechanism for improving health by shifting from focusing on individual behaviors to conceptualizing social determinants of health (SDoH) relevant to the population of interest (Parker, 2017).

## Specifics of the Qualitative Descriptive Approach

Following past community-engaged research with Indigenous peoples (Burnette et al., 2014), we employed a qualitative descriptive methodology for data collection and analysis, which centers the voices of participants in contrast to highly interpretative and abstract methodologies. In qualitative descriptive methodology, data are often collected through semi-structured interviews conducted with individuals or small groups recruited through purposeful sampling (Sandelowski, 2000). Latent concepts are explored through low-level investigation in qualitative description (Sandelowski, 2000; Sullivan-Bolyai et al., 2005). Qualitative description differs from other qualitative research approaches because it relies on low-inference interpretation and description of the data, which contrasts with highly interpretative description approaches (Burnette et al., 2014; Carspecken, 1996; Sandelowski, 2000). Though more interpretive approaches can be useful for understanding health concepts, they can also produce research that is inaccessible to lay audiences (Sullivan-Bolyai et al., 2005). The goal of qualitative description is to produce descriptive findings that have high interpretative validity (Sandelowski, 2000), rather than the production of thick description, theory development (e.g., in the case of grounded theory approaches), or a phenomenological understanding of an experience (Sullivan-Bolyai et al., 2005).

Because qualitative description focuses on understanding the experiences of participants as described in their own words and the understanding and preservation of cultural nuances, it is recommended for research projects with minoritized groups whose views are frequently excluded from dominant culture (Sullivan-Bolyai et al., 2005). Findings are presented as easily understood, detailed recountings of experiences or events in words that resonate with participants themselves (Burnette et al., 2014; Milne & Oberle, 2005; Sandelowski, 2000; Sullivan-Bolyai et al., 2005). Qualitative description is flexible and allows themes to develop inductively while being informed by theory (Sandelowski, 2000; Sullivan-Bolyai et al., 2005).

The internal validity of qualitative descriptive studies is high due to the focus on participant voice and using their own words, in contrast to a reliance on abstract constructions of experiences (Sullivan-Bolyai et al., 2005). Rigor is defined differently for qualitative studies generally and for qualitative descriptive methodology specifically. In qualitative methodology, rigor is dependent on the researchers closely, accurately, and directly representing the views and voices of participants. Toward this goal, we used Milne and Oberele’s (2005) strategies for encouraging high standards of rigor in qualitative studies, including: 1) selection of participants that is methodical but not rigid; 2) encouraging participants to be able to communicate openly and freely; 3) transcripts that are accurate and transcribed verbatim; 4) coding centered on interviewees’ actual word choices and experiences; and 4) having context-driven analysis (i.e., analyzing findings in the context of the specific tribal context). Authenticity was additionally promoted through self-reflection throughout the research process and debriefing with the CAB, colleagues, and the coauthor, who reviewed the hierarchy of themes and respective quotes and the fidelity to the methodology and the culturally congruent process at all times.

The straightforward and low-level interference model used in qualitative description has been suggested as one way to avoid othering the research participant (Hyett et al., 2019; Sullivan-Bolyai et al., 2005). Using a qualitative descriptive approach avoids reliance on an academic or researcher lens, often “culturally incongruent with that of the individual experiencing the health disparity” (Sullivan-Bolyai et al., 2005, p. 129). Qualitative description is considered to be especially well-suited to identifying healthcare barriers and needs for marginalized populations (Sullivan-Bolyai et al., 2005). Because qualitative description strives to create research findings that are easily translatable, particularly in health contexts (Sandelowski, 2000), it was an ideal approach for this project; producing research that has an applied health outcome is important so as not to perpetuate a model of “extractive research” and aligns with Carroll et al.'s (2019) work regarding data for nation rebuilding (Cochran et al., 2008; Smith, 1999). When conducting research with Indigenous communities, the data produced must be of practical use to the community in order to align with the shift toward nation rebuilding in the context of Indigenous data sovereignty (Carroll et al., 2019).

Although not required when using a qualitative descriptive research approach, qualitative descriptive studies can have “hues, tones, or textures” of other qualitative approaches (Burnette et al., 2014, p. 337; Sandelowski, 2000). Our use of qualitative description methodology in this specific context had hues of a life-history approach. Life-history interviews are a form of qualitative research that focuses on portraying events across an individual’s lifespan and facilitates the telling of an individual’s story through their own voice (Creswell & Poth, 2016). Indeed, recommendations to engage in ethical and culturally sensitive research emphasize using appropriate methodology and reinforcing tribes’ strengths, such as storytelling (Burnette et al., 2014); thus, life-history interviews are particularly salient with Indigenous communities. Though this approach can follow the life history of an individual linearly, it is frequently nonlinear, allowing for flexibility and individuals to describe life events as they have experienced and interpreted them (Creswell & Poth, 2007). Indigenous researchers’ previous studies with this specific tribe have also used this method (McKinley et al., 2019).

With its focus on using everyday language and the ability of non-researchers to interpret findings, qualitative description is generally more accessible than many other forms of research and has been used to conduct research in several Indigenous contexts (Bruno et al., 2022; Oster et al., 2014, 2021; Sandelowski, 2000; Sullivan-Bolyai et al., 2005). For example, Oster et al. (2014) noted that qualitative description offers an important lens for non-Indigenous researchers because of the methodology’s emphasis on low interpretation. Using a methodology that emphasizes participant interpretation and understanding of events is also ethically congruent with our theories and principles of cultural sensitivity, cultural humility, and cultural safety. Additionally, incorporating a life-history method into this approach is compatible with the recommendation of Indigenous researchers and scholars for research with Indigenous individuals (Ball, 2005; Denzin et al., 2008; McKinley et al., 2019).

### Community-Based Approach for Selection of Participants

To invite tribal feedback, the lead author reached out to tribal members to create a community advisory board (CAB) comprised of tribal community members. The CAB was key in ensuring all interview questions were relevant and appropriate for women in the tribe, in addition to aiding in participant recruitment and the dissemination of results. The lead author also used snowball sampling to recruit participants. The recruitment flyer was handed out in person by CAB members to potential participants and by the PI to interviewees to recruit additional participants. Inclusion criteria for the study included: 1) self-identifying as a member of the tribe; 2) self-identifying as a woman; and 3) being 18 years of age or older.

We purposefully did not require participants to show proof of enrollment due to the extensive difficulty in proving tribal membership many Indigenous peoples have experienced historically in addition to the highly political — and often exploitative — procedures involved (Cochran et al., 2008). Debates over how best to define community are not simply semantic but can have real consequences for individuals, especially Indigenous groups. Researchers and government agencies generally ignore inter-community differences and treat community categories as distinct and bounded units (Cornwall, 2008). Because of this history, we felt it most ethically appropriate to allow self-identification as a tribal member and did not ask participants to prove or describe their membership — though the lead author did ask how their tribal identity has impacted their lives. All self-identified members of the tribe and women over the age of 18 were eligible to participate in the study. This approach contrasts with approaches where only young or elder women might be interviewed. This range was included to facilitate exploring intergenerational differences in healthcare experiences and to gain a life course perspective.

Researchers, along with public health and social workers, have frequently contributed to the exploitation of Indigenous women (Burnette et al., 2014; Cochran et al., 2008; Creese & Frisby, 2011; Gurr, 2014; Kovach, 2010; Minkler & Wallerstein, 2011; Parker, 2017; Smith, 1999; Strega & Brown, 2015), making the use of community-based and informed research methods especially important (Cochran et al., 2008; Denzin et al., 2008; Nicholls, 2009; Parker, 2017). Because of this history, special precautions were taken before beginning research. Indigenous scholars discuss the importance of research approaches and techniques that are culturally informed, including the need for and importance of having tribal IRBs (Buchanan et al., 2007; Parker, 2017; Saunkeah et al., 2021; Smith, 1999). Tulane University’s IRB approval and tribal council IRB approval were both obtained before recruitment and data collection started. Honoring the autonomy of participants is of the utmost importance considering the long legacy of exploitation experienced by this community and is essential to the guiding theory of cultural safety (Denzin et al., 2008; Israel et al., 2005; Kovach, 2010; Nicholls, 2009; Smith, 1999).

In addition to debriefing with coauthors, the lead author worked closely with their tribal contacts to ensure this project was conducted in a respectful manner and would benefit the tribe. There were no known risks for this study. However, to minimize potential risks, we ensured participation was voluntary and structured the interview process so that participants were in control of when and where we met and conducted interviews. The lead author also encouraged participants to take as long as they needed to consider participating in the study.

### Collaborative Development of the Protocol

Following the recommendation to work with cultural insiders (Burnette et al., 2014; Saunkeah et al., 2021), the protocol for data collection and subsequent semi-structured interview guide was created in partnership with the CAB to conduct individual life-history interviews. The CAB also helped ensure ethical guidelines were followed and the research methodology was appropriate and relevant for women. For example, the tribal liaison suggested that participants may feel more comfortable answering questions about their “romantic relationships” than explicitly asking about sexual relationships. The development of the questions selected for inclusion in the interview guide was informed by previous literature on Indigenous health, history, colonialism, reproductive health, the qualitative description methodological approach, and discussion with tribal community members. Example interview questions included: “What did your family do when you were a child when someone got sick?” and, for women with children, “Where did you give birth?” and “How did the doctors and nurses treat you during labor and delivery?”

Settler-colonial practices continue to perpetuate sexual violence and contribute to disproportionate rates of intimate partner violence (IPV) experienced by Indigenous groups. However, the lead author purposefully did not ask participants about sexual violence or IPV both because it was not a focus of these research questions and in respect of CAB recommendations. Though these topics did not come up in the interviews, the lead author developed a list of resources to address IPV or sexual violence to provide participants if they needed or requested aid. Additionally, the lead author is a master’s-level social worker with experiences in mental health and crisis social work. The authors also have experience working as a crisis counselor at an HIV clinic and with women and children who have experienced sexual violence and abuse. Although the lead author did not provide mental health services to participants, they have experience monitoring and assessing the safety of participants and were able to connect participants to the appropriate resources if necessary or desired. All identifying information was redacted from the transcripts to ensure maximum participant privacy.

To be congruent with the life history overtones of this research approach, the lead author structured interview questions in a way that more closely followed a life-course trajectory (Creswell & Poth, 2016). The interviews flowed naturally, and questions were not always answered or asked in a linear fashion. Life history interviews are a culturally congruent form of data collection and have been used with the tribe previously (McKinley et al., 2019). To ensure that question wording was clear for participants, the lead author piloted the interview guide with two tribal members before beginning interviews. For a full list of interview questions, see Liddell and Kington (2021).

The lead author conducted interviews in individual homes, community centers, and a coffee shop. Previous research has documented the importance of traveling to participants when conducting interviews in contrast to research where the interviewees travel to the researcher (Minkler & Wallerstein, 2011). More specifically, researchers have discussed the significance of traveling to tribal communities when conducting research (Burnette et al., 2014; McKinley et al., 2019). When interviewees were contacted to set up an interview, they were asked where they preferred to be interviewed. Most preferred to be interviewed at a community center (McKinley et al., 2019).

Conducting interviews at this community center allowed for flexibility. Participants could move their interview times if necessary because the lead author would generally spend the entire day at the community center and had a room where they could conduct the interviews in private. It also aided the lead author’s ability to recruit and build rapport with interviewees, as several were employees at the center. Enabling self-determination as to where interviews are held is also congruent with the steps outlined by Burnette et al. (2014) for conducting rigorous and ethical research with Indigenous communities in their *Toolkit for Ethical and Culturally Sensitive Research*.

The lead author followed the responsive interviewing model (Rubin & Rubin, 2005/2012), which focuses on creating a shared understanding between researcher and participant. Responsive interviewing emphasizes flexibility in asking questions and adapting questions to respond to the interview partner and conversation dynamic (Rubin & Rubin, 2005/2012). Interview questions were modified based on the comfort level, knowledge, and experiences of the interviewee and adapted wording as seemed appropriate. Rubin and Rubin (2005/2012) also suggested this model’s responsiveness to participant needs and experiences makes it particularly appropriate when discussing potentially sensitive topics. This model also helps foster the self-determination, because they can tell their story in whatever manner makes the most sense to them. When needed and appropriate, the lead author also guided questions to ensure participants answered all the key research questions. Additionally, the lead author took informal field notes after each interview, meetings with CAB members, and tribal events to aid in their recall, self-reflection, data analysis, and discussion of findings.

Qualitative description is focused on reaching a saturation of themes in the interview process, in contrast to achieving a set number of interviews (Creswell & Poth, 2016; Rubin & Rubin, 2005/2012). Although this approach is flexible, in a review of qualitative descriptive health disparities studies, sample sizes were generally between 20–50 participants (Sullivan-Bolyai et al., 2005), and we aimed to complete interviews within this range. The lead author conducted life-history interviews with 31 participants. Saturation was achieved at 27 interviews, but the remaining interviews were conducted to further verify saturation, because the interviews had already been scheduled, and because the lead author desired to keep their agreements with tribal members.

After completion of a conventional qualitative content analysis, the findings from this study were shared at tribal council meetings and with study participants through member checks. Participants were mailed or emailed a summary of findings, based on their preference. The lead author followed up with all participants who consented to participate in member checks, during which they provided a summary of study results. Only one participant declined to be contacted following their interview. Two additional participants could not be contacted because the provided email addresses were invalid when member checks were sent.

Though most participants requested results be emailed to them, four participants requested results be mailed, and a printed copy of the findings was sent to these interviewees. The lead author reached out to participants with a summary of results two to four times. No respondents requested changes to results. As part of the research agreement with the tribe, the lead author agreed to present a summary of the research findings to the tribal council before publishing any findings from the research. An initial presentation in February 2020 was postponed due to weather, and in May 2020, we presented these findings virtually due to COVID-19 safety concerns to the tribal governing council. The second author, based on their work with the focal tribe, introduced and attended this presentation. All tribal governing council members were provided a summary of research findings before the meeting and were invited to ask questions after the 20-minute presentation of these findings. Tribal council members were supportive of the project and expressed interest in having us present these findings during a full tribal council meeting at a later date. We also agreed not to identify the tribe in publications and to share any resulting publications with the tribal archivist.

In the findings, we give interviewee quotes anonymous identifiers (IDs) to demonstrate how themes are represented across participants while protecting confidentiality. Though some researchers provide participants with pseudonyms instead of using ID numbers, this practice has been critiqued because the process of using and creating pseudonyms is not neutral and can feel inauthentic to some participants (Allen & Wiles, 2016; Corden et al., 2006). We followed the practices recommended by the CAB and those previously used in research with this tribe (McKinley et al., 2019). Unfortunately, it is beyond the scope of this article to fully describe all findings. The results shared next are representative of what we feel is important to highlight for this article. For a summary of key themes, please see Liddell and McKinley (2022).

## Iterative Process of Reflection and Action in CBR

The lead author’s role as a researcher, the role of researchers in general, and the impact of research with Indigenous peoples came up in some interviews. The lead author’s goal was to be as transparent as possible about research goals and frameworks used in designing this study. One participant described appreciating the lead author connecting the conversation about community strengths and values with individual health: “Yeah, like you said, it’s all related. I’m glad you recognize that because that’s something really hard to explain in academia because, it’s like, it seems like I’m all over the place, but I’m not.” She noted in this quote her impression that researchers often tend to view issues dichotomously and not see the relationship between issues or concepts. This participant also expressed her concern that she would often find articles published about the tribe where findings had not been shared or presented to tribal members, nor had the published article even been sent to the tribe: “Like when I’m like researching online or something and I’ll see like this article, like, ‘Hey, has anyone ever seen this article?’ And they’re [other tribal members] like, ‘Who? What is that?’” The lead author expressed their commitment to sharing all published articles from this research with tribal council or tribal agency staff, in addition to discussing the process they would use to share and present findings to tribal members after completing data analysis.

In another instance, this same participant described a researcher who studied the number of environmental toxins in community members’ blood and, again, never shared the findings with tribal members. That this previous researcher had not shared their findings was especially concerning to this participant because one tribal member had been an important community liaison for this researcher and had been instrumental in organizing community members around environmental justice issues and increasing study participation:

[The data] It is five books … I was talking to her [community liaison] about it one day and she’s like, “Yeah, I’ve never seen them.” … I was like, “Are you … kidding me? You literally gave her blood for like a year and you gave her like kids in the communities’ blood, blood for like a year and you’ve never seen them?” She’s like, “No, she [the researcher] never bought them back.” … And they [researchers] just kind of come, they publish and then they go away.

Another woman expressed hope that more researchers would use their results to inform actual projects and interventions in the community: “Yeah, just don’t let it die. Because that’s what happens lots of times. That’s exactly what happens. … There’s a lot of aspiration about it but then after that it… Keep the fire burning.” Participants were often quite interested in what prompted the lead author to be interested in working with the tribe and how the lead author viewed themselves as a researcher. Here is an example of this conversation with another participant:

[Participant]: I hope this all goes well, and, or you are working towards your PhD?

[PI]: Exactly … I work with [advisor name], so she, she’s my … adviser … so that was kind of how I got introduced to people here and then, then I just kind of fell in love with everyone here and started working with [CAB member, name omitted].

[Participant]: Right. [laughs].

[PI]: I have a public health and the social work background and then, um, I study women’s health … I wanted to do this in part is because I really want to make the, two talk to each other cause I feel like social workers are really good at working with people. Doctors know the medicine, but we need to find a way that—

[Participant]: To get them together.

[PI]: Yeah, exactly. So that, um, that’s kind of my interest. And then, you know, I’m really passionate about women’s health.

This excerpt is similar to how the lead author described their interest in the project with other participants who asked. Clarity and authenticity on the part of the lead author were important elements of the interview process and are reflected in this excerpt.

## Indigenous-Centered Research for Relationship-Building

The findings are representative of an overall research environment that allows for the honest discussion of the research context, an important element of meaningful reflexivity (Dodgson, 2019; Folkes, 2023). For example, in describing past oppressive research practices and the lack of data sharing by other researchers, participants are sharing important background in the study setting. These excerpts demonstrate a mutual curiosity between interviewee and interviewer and suggest a breaking down of the historical binary of “researcher” and “researched” (TallBear, 2014). Expanding this historical dichotomy positions the interviewer and interviewee as knowledge bearers and receivers (TallBear, 2014).

Reflexivity was integrated throughout the study. The lead author maintained a journal throughout to document their thoughts and reflections. The lead author was also fluid and adaptable in their approach to the project, welcomed CAB feedback, and adapted the project as requested. In the interviews and during analysis, the lead author strove to be responsive to participants’ needs. For example, when tribal members desired to also talk about elder or community help during interviews, the lead author did not attempt to redirect the interviewee back to questions specific to sexual and reproductive health. Instead, the lead author provided time and space to discuss these topics, which interviewees view as related and valuable.

The lead author’s desire to conduct research that is of real benefit to marginalized communities has been essential for shaping this research approach, the lead author’s agenda for this project, and her career as a scholar. The lead author takes a holistic and interdisciplinary approach, focusing on reproductive health research developed with the community because of the problematic history of research and health practitioner exploitation of marginalized communities.

TallBear (2014) urged researchers to consider the research process with Indigenous communities as a relationship-building process and an opportunity to share knowledge, not the sole purpose of collecting data. Through this study and additional research conducted alongside this tribe, relationships between the authors and the tribe were formed and strengthened. To conduct this research collaboratively and build rapport and relationship, we engaged with tribal members throughout the process, demonstrated respect for the community, and facilitated community autonomy (Buchanan et al., 2007; Parker, 2017). The research project was developed in conjunction with members of the tribe, and we received tribal approval to conduct this research. Additionally, Kovach (2010) has argued Indigenous-centered research should have a strong focus on meaningful description in its methodology and as part of its presentation of methods and findings, which is a central component of qualitative description and life-history ethnography and are methods employed in this study described in more detail (Sandelowski, 2000; Sullivan-Bolyai et al., 2005). This research approach also emphasizes the importance of localizing findings and putting them in context for the specific tribe in this study. This approach ultimately makes these findings more useful for tribal members, although it may reduce generalizability. These findings have been shared with all participants, in addition to tribal leadership, and may be used to develop interventions for tribal members. Not all research methodologies allow for research products that are as easily used by non-researchers.

Because of the authors’ previous work with the tribe, the lead author’s rapport with the CAB members, and the fact that tribal council support was secured before conducting this research, we experienced relatively few barriers in conducting this study. We were able to interview enough women to achieve saturation in research themes and to interview women across a range of ages. We were successful in large part because of the support of the CAB members and because of the lead author’s success in early interviews, as later interviewees were often referred to us by previous interviewees. We achieved these research goals because of our strong attention to our own positionality and power dynamics in the research relationship and our own theoretical and philosophical views about conducting meaningful and culturally congruent research. Although following the research strategies outlined in Table 1 is time-intensive, it produces rich and relevant data acquired in an ethical way.

An additional strength of this research approach is that it allowed for the emergence of unexpected themes. The semi-structured interview guide — in addition to following the responsive-interviewing model — encouraged participants to describe in their own words what was important to them and what they wanted us to know about. Although data specific to women’s reproductive and sexual health experiences described in this project are extremely rich, an additional wide range of themes women identified related to community health problems, needed programs, and general healthcare experiences are also relevant for addressing health disparities more broadly. Although beyond the initial scope of this research project (identifying supports and barriers in women’s sexual and reproductive health), these findings will be used to inform future publications about these important topics. Research that uses culturally congruent and sensitive methods not only increases the rigor of the research, it is also more likely to create knowledge that is useful for both the community and the investigator (Burnette et al., 2014; Minkler & Wallerstein, 2011; Parker, 2017; Smith, 1999; TallBear, 2014).

## Conclusion

In this article, we magnified best practices for conducting community-engaged research by exemplifying a specific study on the reproductive and sexual health experiences of Indigenous women. We hope future researchers can use this approach as a template for conducting culturally congruent, rigorous, and high-quality research on Indigenous health topics. Although following this methodological approach and the steps outlined in the Burnette et al. (2014) toolkit can be resource intensive, researchers have an obligation to invest time and resources when conducting research with Indigenous groups. This cultural sensitivity is particularly important when addressing nuanced topics, such as reproductive and sexual health. In this article, we provide a discussion of the importance of researcher positionality and the lead author’s own self-reflection in completing this study. We describe the methodological approach — qualitative description with life-history overtones — including an overview and description of this approach and why it was appropriate for this study. We discuss using semi-structured in-depth interviews for data collection. We also offer a critique of conventional research methods and describe how we attempt to use research practices that are ethical and culturally congruent. Following the strategies outlined in this article may ultimately produce ethical research, resulting in interventions that are truly useful for tribal members because their voices are central throughout the entire process.

## Figures and Tables

**Table 1.** Implementation Strategies from Author(s) (2014) “Toolkit of Strategies for Culturally Sensitive and Ethical Research with AI/AN Communities”

Strategy	Implementation

Become Educated	The PI has studied Indigenous issues throughout their PhD program, both nationally and with the focal tribe, and strives to continually learn more.
Work with a Cultural Insider	The PI worked with a CAB to design the study protocol and interview guide.
Get Invited	The PI was invited to do this project after conversations with one of the CAB members about community needs.
Exhibit Cultural Humility	The PI goes into this work being open to changes, challenges, and any criticism from tribal members about this research, and with a genuine desire that this work be beneficial to tribal members.
Be Transparent	The PI has been open with tribal members about any potential uses of the research findings and any research funds that are available and their use.
Spend Time in the Community	The PI has interacted with community members over many years and has traveled to the community for tribal council events, to meet with tribal members, and to conduct interviews.
Collaborate	The PI worked with the CAB to develop the project and interview guides, and to discuss potential uses of the findings to develop interventions in the community.
Listen	The PI attended tribal council meetings and events and sought feedback from the CAB and interviewees about this project and its potential uses.
Build a Positive Reputation	The PI has developed a positive reputation through their relationship with one of the CAB members and through working with the tribal community via coauthor. They have attended tribal council meetings and have kept promises they have made to tribal members about this research, such as presenting these findings to the tribal council and following up with interviewees to share findings.
Commit Long-Term	The PI is committed to having these research findings be of use to tribal members and will continue to present these findings at community events as the tribe desires going forward and to see through any interventions that emerge from this research.
Use a Memorandum of Understanding (MOU)	The PI worked with the tribal governing council, the CAB, and coauthor to create a research agreement document which was presented at a tribal council meeting and received tribal council approval before conducting any research.
Use a Cultural Reader	The CAB provided feedback and reviewed the study protocol, interview guide, and the summary of research findings before they were presented to the tribal council and sent out to interviewees for member checks.
Go the Distance	The PI traveled to the tribe to meet with CAB members, attend council meetings and to conduct interviews where participants desired.
Demonstrate Patience	The PI followed tribal council protocol and waited to begin research until they had received their approval and followed the CAB’s timeline for beginning research activities.
Enable Self-Determination	The PI enabled participant self-determination by allowing participants to decide where they wanted to have interviews conducted. The PI used a flexible interview guide that followed a life-history trajectory as this was culturally congruent and allowed participants to decide what to answer and how.
Use a Tribal Perspective	Critical theory, life-course, eco-systemic, resilience, and reproductive justice frameworks and approaches were used in this study. These approaches are strength-based and culturally congruent, and center the voices of Indigenous participants.
Use Appropriate Methodology	Qualitative Description with hues of life-history was used which is culturally congruent with work with Indigenous peoples.
Reinforce Cultural Strengths	Focusing on the strengths and resilience of tribal members was a focal part of this project. The interview guide was designed to elicit strengths and resilience and results were analyzed to center cultural strengths.
Honor Confidentiality	The identity of this tribe will be kept confidential in all publications and conference presentations and this dissertation will not be made public.
Advocate	The PI has tried to use their platform as a university researcher to advocate for Indigenous issues and am committed to continuing to do this throughout their career.
Reciprocate and Give Back	The PI compensated interviewees for their time with gift cards and has committed to continuing to share research findings with tribal members at community events and allowing and assisting with the use of these findings in tribal grant applications or reports as the tribe desires, in addition to sharing any resulting publications with tribal members. It is also hoped that these findings might inform the development of future intervention.
Allow for Fluidity and Flexibility	Interviews took place at the times and locations participants preferred, and a flexible interview guide was used to facilitate participant self-determination.
Develop an Infrastructure	The PI created a CAB and have sought their feedback in all aspects of this project and in the development of any future interventions.
Invest Resources	The PI invested resources in the form of gift cards for community members who participated.

*Note.* Table adapted from Burnette et al., (2014) “A toolkit for ethical and culturally sensitive research: An application with Indigenous communities.”
